# The age‐dependent association between aortic pulse wave velocity and telomere length

**DOI:** 10.1113/JP273689

**Published:** 2017-01-24

**Authors:** Barry J. McDonnell, Yasmin, Lee Butcher, John R. Cockcroft, Ian B. Wilkinson, Jorge D. Erusalimsky, Carmel M. McEniery

**Affiliations:** ^1^ Cardiff School of Health SciencesCardiff Metropolitan UniversityCardiffUK; ^2^ Division of Experimental Medicine & ImmunotherapeuticsUniversity of CambridgeCambridgeUK; ^3^ Division of Cardiology, New York‐Presbyterian HospitalColumbia UniversityNew YorkNYUSA

**Keywords:** ageing, aortic stiffness, telomere Length

## Abstract

**Key points:**

- Age significantly modifies the relationship between aortic pulse wave velocity and telomere length.
- The differential relationships observed between aortic pulse wave velocity and telomere length in younger and older individuals suggest that the links between cellular and vascular ageing reflect a complex interaction between genetic and environmental factors acting over the life‐course.

**Abstract:**

Ageing is associated with marked large artery stiffening. Telomere shortening, a marker of cellular ageing, is linked with arterial stiffening. However, the results of existing studies are inconsistent, possibly because of the confounding influence of variable exposure to cardiovascular risk factors. Therefore, we investigated the relationship between telomere length (TL) and aortic stiffness in well‐characterized, younger and older healthy adults, who were pre‐selected on the basis of having either low or high aortic pulse wave velocity (aPWV), a robust measure of aortic stiffness. Demographic, haemodynamic and biochemical data were drawn from participants in the Anglo‐Cardiff Collaborative Trial. Two age groups with an equal sex ratio were examined: those aged <30 years (younger) or >50 years (older). Separately for each age group and sex, DNA samples representing the highest (*n* = 125) and lowest (*n* = 125) extremes of aPWV (adjusted for blood pressure) were selected for analysis of leukocyte TL. Ultimately, this yielded complete phenotypic data on 904 individuals. In younger subjects, TL was significantly shorter in those with high aPWV *vs*. those with low aPWV (*P* = 0.017). By contrast, in older subjects, TL was significantly longer in those with high aPWV (*P* = 0.001). Age significantly modified the relationship between aPWV and TL (*P* < 0.001). Differential relationships are observed between aPWV and TL, with an inverse association in younger individuals and a positive association in older individuals. The links between cellular and vascular ageing reflect a complex interaction between genetic and environmental factors acting over the life‐course.

## Key points

- Age significantly modifies the relationship between aortic pulse wave velocity and telomere length.
- The differential relationships observed between aortic pulse wave velocity and telomere length in younger and older individuals suggest that the links between cellular and vascular ageing reflect a complex interaction between genetic and environmental factors acting over the life‐course.

**Abbreviations**
aPWV: aortic pulse wave velocity
BMI: body mass index
CRP: C‐reactive protein
CV: cardiovascular
HDL: high‐density lipoprotein
MAP: mean arterial pressure
T/S: telomere/standard
TL: telomere length

## Introduction

With ageing, large arteries stiffen as a consequence of elastin degradation and/or vascular remodelling (London, 2001; Yasmin *et al*. 2005), which manifests as an increase in aortic pulse wave velocity (aPWV) throughout life (McEniery *et al*. 2005). Therefore, aPWV has become a widely accepted measure of vascular age. Traditional cardiovascular (CV) risk factors (e.g. total cholesterol, glucose, physical inactivity) and inflammation are associated with increased aPWV (Mora *et al*. 2007; McEniery *et al*. 2008; McDonnell *et al*. 2013) and aPWV is now recognized as an important, independent determinant of CV disease progression (Safar *et al*. 2003) and events (Ben‐Shlomo *et al*. 2014). However, although the severity and life‐course of exposure to CV risk factors increase over time, these factors do not account for all of the risk associated with vascular ageing, suggesting that other mechanisms must be involved.

In recent years, shortening of telomeric DNA has been linked with biological ageing (Samani & van der Harst, 2008; Huzen *et al*. 2010). Telomeres are specialized DNA protein complexes located at both ends of each chromosome and function as chromosomal caps preventing genetic instability and cellular senescence (Karlseder *et al*. 2002; Artandi, 2006). With increasing age and number of cell divisions, progressive shortening of telomere length (TL) leads to a point of cellular senescence, suggesting that TL provides a measure of cellular age (Blasco, 2005). Two recent meta‐analyses have demonstrated that telomere shortening is related to CV (Haycock *et al*. 2014) and metabolic outcomes (D'Mello *et al*. 2015), although there was significant heterogeneity in observations within these studies. Interestingly, previous studies have also reported significant cross‐sectional associations between TL and measures of vascular stiffness (Benetos *et al*. 2001; Nawrot *et al*. 2010; Wang *et al*. 2011) and a recent longitudinal study illustrated that a faster rate of telomere shortening was associated with increased carotid intima‐media thickness, independent of traditional CV risk factors (Masi *et al*. 2014), leading to the hypothesis that telomere shortening provides a cellular and genetic link to vascular ageing. However, observational studies conducted mostly in middle‐aged and older adults have shown weak or no relationship between TL and chronological age, vascular stiffness or CV disease (Tentolouris *et al*. 2007; De Meyer *et al*. 2012; Morgan *et al*. 2014).

In the present study, we explored the relationship between TL and aortic stiffness in two distinct groups of healthy individuals; those aged less than 30 years and those over 50 years, who were pre‐selected on the basis of having aPWV in the lower or upper extremes for their age group and sex. We hypothesized that higher aPWV is associated with shorter TL in healthy individuals.

## Methods

### Study population

**Figure 1:**
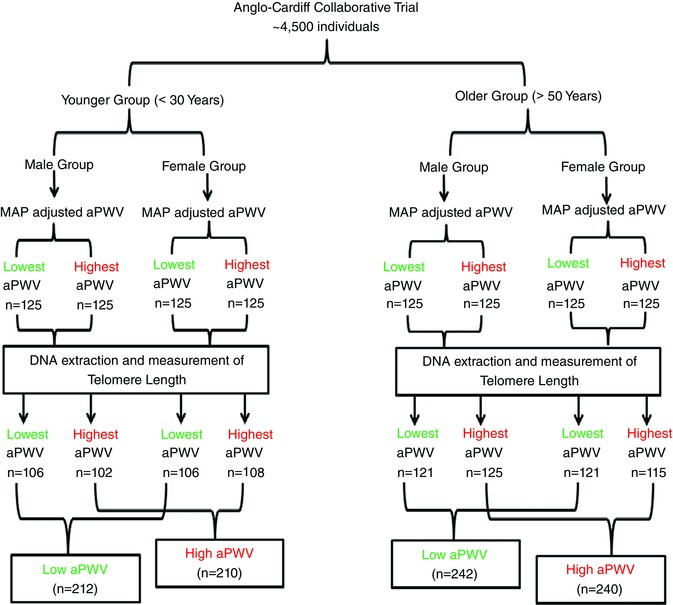
Group stratification and classification [Color figure can be viewed at wileyonlinelibrary.com]

Data on men and women, where complete information concerning demographic, haemodynamic and biochemical characteristics were available, were drawn from the Anglo‐Cardiff Collaborative Trial (McEniery et al. 2005), which consists of approximately 4500 individuals with detailed phenotypic data. Two age groups were examined: those aged <30 years (younger) or >50 years (older). Because aPWV is closely related to the mean arterial pressure (MAP; higher MAP = higher aPWV), aPWV was adjusted for MAP using linear univariate regression models, separately for each age group and sex, after determining that analyses based on linear models were the most appropriate for each group. This approach ensured that we examined associations between TL and intrinsic aortic stiffness, rather than simply selecting samples for TL analysis based on a proxy for blood pressure. Samples were then selected on the basis of the MAP‐adjusted aPWV of an individual being located in either ‘extreme’ of the aPWV distribution (i.e. upper or lower 15%), which equated to the highest (*n* = 125) and lowest (*n* = 125) aPWV values for each age‐group and sex. This yielded samples from 1000 healthy individuals, who were free from CV disease and medication, and who had undertaken a detailed lifestyle and medical history questionnaire (Fig. 1). The study complied with the *Declaration of Helsinki* and ethical approval was obtained from the Local Research Ethics Committees, with written informed consent being provided by all participants.

### Protocol

Height and weight were assessed and a medical history questionnaire, including details of medication was completed. Following 15 min of supine rest, brachial blood pressure and carotid and femoral artery pressure waveforms were recorded, and aPWV determined. Blood samples (20 mL) were then drawn from the antecubital fossa. Serum, plasma and buffy coat samples were then obtained and stored at −80 °C for subsequent analyses. Cholesterol, triglycerides, glucose and C‐reactive protein (CRP) were determined using a standard methodology in an accredited laboratory.

### Haemodynamic measurements

Brachial blood pressure was measured with the participant resting supine, using a validated semi‐automated oscillometric device (HEM‐705CP; Omron Corporation, Kyoto, Japan), in accordance with the British Hypertension Society guidelines (O'Brien *et al*. 1996). All measurements were taken in duplicate and mean values were used in the subsequent analyses. aPWV was measured using the SphygmoCor system (Atcor Medical, Sydney, Australia) by sequentially recording ECG‐gated carotid and femoral artery waveforms, as previously described in detail (Wilkinson *et al*. 1998). Path length for the determination of aPWV was measured, using a tape measure, as the surface distance between the supra‐sternal notch and femoral site minus the distance between the supra‐sternal notch and carotid site.

### DNA extraction, quantification and assessment of TL

DNA was extracted from the buffy coats using a commercially available GeneCatcher kit (Invitrogen, Paisley, UK) and then quantified and checked for purity using a Nanodrop ND‐1000 spectrophotometer (Labtech, Uckfield, UK). DNA quality was assessed by the absorbance ratio at 260 and 280 nm, and only samples with a ratio between 1.8 and 1.9 were included in the study. In addition, the integrity of the DNA samples was confirmed by gel electrophoresis in 50 randomly selected samples. Leukocyte relative mean TL was measured as the telomere/standard (T/S) ratio in triplicate by a monochrome multiplex quantitative PCR assay, using a Bio‐Rad CFX96™ RT‐PCR detection system (Bio‐Rad, Hemel Hempstead, UK) as described previously (Steptoe *et al*. 2011). TL is expressed as the ratio of the telomeric DNA signal to the signal of the single copy gene β‐globin, used as an internal standard (T/S ratio) (Cawthon, 2009). There was very little variability between the triplicate readings (<2%) in samples from the two study groups, confirming the good reliability of the quantitative PCR assays.

### Statistical analysis

All data were analysed using SPSS, version 20.0 (IBM Corp., Armonk, NY, USA). Two‐way ANOVA (age × aPWV) was used to investigate the relationship between aPWV and haemodynamic and biochemical variables, including the T/S ratio. A test for interaction was used to determine whether age modified any of these associations, with planned contrasts used to examine differences between aPWV groups for younger and older individuals, respectively. A modified Bonferroni correction was applied to adjust for multiple comparisons. Pearson correlation coefficients were used to determine bivariate correlations between variables. Finally, within each age group, multiple regression analyses based on the enter method were performed to determine those parameters remaining independently associated with aPWV, after including variables traditionally known to confound measurements of aPWV (McEniery *et al*. 2010). Linear models were chosen after establishing that these provided similar associations with age compared to non‐linear approaches. Log‐transformed T/S ratio values were used for all analyses because the T/S ratio was non‐normally distributed. Unless otherwise stated, the results are expressed as the mean ± SD. *P* < 0.05 was considered statistically significant.

## Results

In total, 96 samples were excluded from the analysis because of poor quality or insufficient DNA to enable accurate detection of TL, yielding 422 young participants (212 with low aPWV and 210 with high aPWV) and 482 older participants (242 with low aPWV and 240 with high aPWV) in whom complete data were available (Fig. 1).

### Anthropometric characteristics

Anthropometric characteristics are presented in Table 1. In the younger participants, only weight and body mass index (BMI) were significantly higher in those with high *vs*. low aPWV (*P* = 0.013 and *P* = 0.016, respectively). However, in older participants, weight, BMI, smoking status and pack years were all significantly higher in those with high *vs*. low aPWV (*P* < 0.001, *P* = 0.001, *P* = 0.026 and *P* = 0.002, respectively). Age significantly modified the relationship between aPWV and smoking pack years (*P* = 0.017).

**Table 1:** Comparisons and interactions between demographic, haemodynamic and biochemical variables for low and high aPWV groups in younger and older individuals

	Younger		Older			
	Low	High	Low	High	Overall ANOVA	Interaction
Parameter	aPWV	aPWV	aPWV	aPWV	*P* value	*P* value
*n*	212	210	242	240		
Age (years)	20 ± 4	20 ± 2	68 ± 7	67 ± 7	<0.001	0.060
Sex (% male)	49	49	50	50	0.983	0.869
Height (m)	1.7 ± 0.1	1.7 ± 0.1	1.7 ± 0.1	1.7 ± 0.1	<0.001	0.904
Weight (kg)	67.3 ± 13.6	70.5 ± 13.4*	72.1 ± 14.1	76.7 ± 14.5***	<0.001	0.436
BMI (kg m^–2^)	22.7 ± 3.4	23.5 ± 3.5*	25.9 ± 4.2	27.1 ± 4.5**	<0.001	0.336
Smokers (%)	10.1 ± 30.1	15.0 ± 35.8	8.6 ± 26.5	13.7 ± 34.5*	<0.001	0.776
Smokers (pack years)	1.0 ± 3.1	1.4 ± 3.3	4.1 ± 8.0	6.7 ± 10.2**	<0.001	0.017
Brachial SBP (mmHg)	116 ± 13	115 ± 13	139 ± 17	140 ± 20	<0.001	0.324
Brachial DBP (mmHg)	68 ± 8	68 ± 8	81 ± 9	78 ± 9**	<0.001	0.051
Brachial PP (mmHg)	49 ± 12	48 ± 11	58 ± 13	62 ± 15**	<0.001	0.010
Brachial MAP (mmHg)	82 ± 9	81 ± 9	102 ± 11	100 ± 13	<0.001	0.717
HR (beats min^–1^)	67 ± 12	68 ± 12*	64 ± 9	70 ± 10***	<0.001	0.001
aPWV (m s^–1^)	4.6 ± 0.4	6.8 ± 0.8***	7.3 ± 1.2	12.4 ± 2.2***	<0.001	<0.001
Adj aPWV (m s^–1^)	4.6 ± 0.4	6.9 ± 0.5***	6.7 ± 0.8	12.1 ± 1.6***	<0.001	<0.001
Total cholesterol (mmol l^–1^)	3.94 ± 0.83	4.16 ± 0.88**	5.65 ± 1.11	5.54 ± 1.08	<0.001	0.011
HDL (mmol l^–1^)	1.40 ± 0.40	1.49 ± 0.40*	1.61 ± 0.39	1.49 ± 0.43**	<0.001	<0.001
LDL (mmol l^–1^)	2.16 ± 0.70	2.23 ± 0.76	3.49 ± 0.92	3.35 ± 0.94	<0.001	0.059
Triglycerides (mmol l^–1^)	0.96 ± 0.67	1.10 ± 0.07	1.29 ± 0.64	1.49 ± 0.43***	<0.001	0.001
Glucose (mmol l^–1^)	4.81 ± 0.97	4.78 ± 1.68	5.09 ± 2.86	5.17 ± 1.38	0.067	0.656
CRP (mg l^–1^)	2.25 ± 3.45	2.27 ± 2.83	2.67 ± 3.91	4.17 ± 6.05**	0.001	0.169
Log‐transformed T/S ratio	0.100 ± 0.118	0.076 ± 0.086*	0.012 ± 0.111	0.052 ± 0.099***	<0.001	<0.001

SBP, systolic blood pressure; DBP, diastolic blood pressure; PP, pulse pressure; Adj aPWV, aortic pulse wave velocity adjusted for mean arterial pressure; LDL, low‐density lipoprotein. Overall ANOVA represents the results of two‐way ANOVA. Interaction represents the age × aPWV interaction term. ^*^ *P* < 0.05, ^**^ *P* < 0.01 and ^***^ *P* < 0.001 *vs*. the Low aPWV group.

### Haemodynamic indices

**Figure 2:**
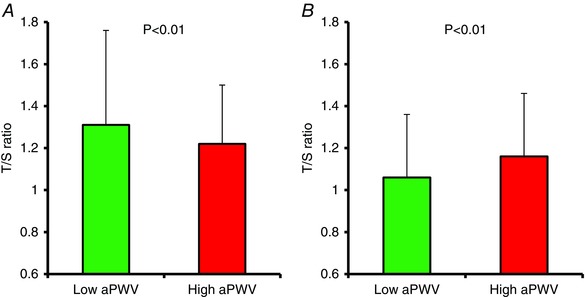
Difference in T/S ratio between the Low and High aPWV groups *A*, younger individuals. *B*, older individuals. [Color figure can be viewed at wileyonlinelibrary.com]

Haemodynamic indices are presented in Fig. 2 and Table 1. No significant differences were observed between younger participants with low *vs*. high aPWV. By contrast, in older participants, brachial diastolic blood pressure was significantly lower and pulse pressure and heart rate were significantly higher in those with high aPWV (*P* = 0.002, *P* = 0.004 and *P* < 0.001, respectively). Age significantly modified the relationship between aPWV and brachial pulse pressure and heart rate (*P* = 0.010 and 0.001, respectively).

### Biochemical indices

Total and high‐density lipoprotein (HDL) cholesterol were both significantly higher in younger participants with high aPWV *vs*. those with low aPWV (*P* = 0.007 and *P* = 0.018, respectively). By contrast, HDL was significantly lower (*P* = 0.002) and triglycerides and CRP significantly higher (*P* < 0.001 and *P* = 0.001, respectively) in older participants with high aPWV *vs*. those with low aPWV. Age significantly modified the relationship between aPWV and total cholesterol, HDL cholesterol and triglycerides (*P* = 0.011, *P* < 0.001 and *P* = 0.001, respectively).

### Telomere length

As hypothesized, the T/S ratio was significantly lower in young participants with high aPWV *vs*. those with low aPWV (*P* = 0.017). By contrast, the T/S ratio was significantly higher in older participants with high aPWV *vs*. those with low aPWV (*P* < 0.001). Age significantly modified the relationship between aPWV and T/S ratio (*P* < 0.001). Treating the data as continuous variables, aPWV was inversely correlated with T/S ratio (*r* = −0.12, *P* = 0.013) in younger participants but positively correlated with T/S ratio in older participants (*r* = 0.20, *P* < 0.001). In multiple regression analyses, after entering traditional confounding factors, the T/S ratio remained independently associated with aPWV in both younger and older participants. However, the association remained inverse in younger participants (Table 2) but positive in older participants (Table 3).

**Table 2:** Multiple regression analysis illustrating the parameters associated with aPWV in younger individuals

Dependent variable: aPWV	Unstandardized β	SE	Standardized coefficients (β)	*t*	*P* value
Age	0.073	0.240	0.147	2.997	0.003
Sex	−0.221	0.121	−0.089	−1.822	0.069
Mean arterial pressure	0.019	0.008	0.135	2.557	0.011
Heart rate	0.013	0.005	0.126	2.472	0.014
BMI	0.035	0.019	0.094	1.895	0.059
Smoking status	0.194	0.106	0.087	1.822	0.069
T/S ratio	−1.375	0.581	−0.113	−2.368	0.018

**Table 3:** Multiple regression analysis illustrating the parameters associated with aPWV in older individuals

Dependent variable: aPWV	Unstandardized β	SE	Standardized coefficients (β)	*t*	*P* value
Age	0.088	0.019	0.199	4.702	<0.001
Sex	−0.287	0.276	−0.046	−1.217	0.299
Mean arterial pressure	0.048	0.011	0.181	4.158	<0.001
Heart rate	0.087	0.013	0.284	6.557	<0.001
BMI	0.091	0.031	0.128	2.912	0.004
Smoking status	0.161	0.148	0.048	1.090	0.276
T/S ratio	4.709	1.289	0.160	3.653	<0.001

## Discussion

The present study is the first to illustrate that age modifies the association between TL and aPWV in healthy individuals, with an inverse association present in younger individuals and a positive association present in older individuals. These data suggest that the links between cellular and vascular ageing reflect a complex interaction between genetic and environmental factors acting over the life‐course.

Our data are in agreement with the majority of published studies showing that shorter TL is associated with increased chronological age (Blasco, 2005). TL is also considered to be significantly affected by genetics, (Brouilette *et al*. 2008; Samani & van der Harst, 2008), together with psychosocial and environmental factors such as hostility (Brydon *et al*. 2012), educational attainment (Steptoe *et al*. 2011), sleep duration (Jackowska *et al*. 2011) and oxidative stress (Kiecolt‐Glaser *et al*. 2013; Tarry‐Adkins & Ozanne, 2014). Moreover, TL has been linked with both metabolic and CV outcomes in recent meta‐analyses (Haycock *et al*. 2014; D'Mello *et al*. 2015), albeit with significant heterogeneity between observations within these studies. Interestingly, recent data also suggest that both genetic and environmental factors affect large artery stiffness (McEniery *et al*. 2008; Yasmin & O'Shaughnessy, 2008; Mitchell *et al*. 2012; McDonnell *et al*. 2013) and a recent large meta‐analysis demonstrated that aPWV independently predicts stroke and CV outcomes (Ben‐Shlomo *et al*. 2014). However, the links between cellular and vascular ageing are not well understood, and merit further investigation.

A recent longitudinal study illustrated that a faster rate of telomere shortening is associated with increased carotid intima‐media thickness (Masi *et al*. 2014) in middle‐aged individuals, indicating that accelerated cellular ageing is associated with early atherosclerosis. Furthermore, cross‐sectional studies have shown that a shorter TL is associated with measures of vascular stiffness, including increased aPWV and reduced carotid distensibility (Benetos *et al*. 2001; Nawrot *et al*. 2010; Wang *et al*. 2011). However, other cross‐sectional studies have reported no association between TL and arterial stiffness in females (Benetos *et al*. 2001) and in diabetics (Tentolouris *et al*. 2007). Unfortunately, all of these studies contained relatively few subjects, with significant associations between TL and stiffness being reported in males only. Moreover, the studies included predominantly older adults, in whom the degree of life‐course exposure to cardiovascular risk factors probably differed significantly between individuals.

In the present study, we examined distinct groups of younger and older individuals, selected from the extremes of the aPWV distribution. This approach allowed us to explore the association between cellular and vascular ageing with sufficient power. However, we attempted to minimize the confounding influence of blood pressure on aPWV by selecting samples for analysis of TL based on aPWV values that had been adjusted for blood pressure. The significant, inverse association between aPWV and TL observed in younger individuals suggests that cellular and vascular ageing may be linked in these individuals, with TL remaining independently associated with aPWV in multiple regression analyses. Aortic stiffness is relatively homogeneous in younger individuals, with a smaller spread of values and a SD from the mean of approximately half that seen in individuals aged >50 years (McEniery *et al*. 2005). This homogeneity may reflect the limited exposure to cardiovascular and other risk factors in younger, compared to older, individuals. Nevertheless, meaningful differences in aPWV can still be observed in younger subjects, especially when extremes of the distribution are considered, as in the present study. Indeed, based on our data, a difference of 0.09 in T/S ratio was equivalent to a difference in vascular age of approximately 20 years, according to the spread of aPWV values for the whole group.

By contrast, although aortic stiffness is greater in older individuals, it is much more heterogeneous, which most probably reflects variations between individuals in life‐course environmental exposures, and their interaction with genetics and/or early life factors. As such, the positive association between TL and aPWV observed in older individuals in the present study argues against any direct link between cellular and vascular ageing in these individuals. Instead, it may be that associations between cellular and vascular ageing in certain individuals are influenced by exposure to common (or different) risk factors, or that different processes drive different biological markers of ageing in older individuals. Alternatively, recent data suggest that individuals with longer TL at younger ages tend to show a greater rate of TL shortening with age (Muezzinler *et al*. 2013; Weischer *et al*. 2014), which may account for some of our findings. Interestingly inter‐individual differences in TL are assumed to be established early in life (Daniali *et al*. 2013) and may partly determine individual trajectories of TL shortening over the life‐course. However, our data in older individuals are probably influenced by a healthy survivor effect. That is, individuals with high aPWV and short TL may have been selected out of the analysis because of the presence or a history of CV disease and events. Nevertheless, the current data in older individuals may explain, in part, the heterogeneity between TL and CV outcomes (Weischer *et al*. 2014; D'Mello *et al*. 2015) and cerebrovascular disease (Haycock *et al*. 2014) reported previously and raise the question of whether TL alone should be used as a marker of CV risk in older individuals because many factors decrease or even increase TL, independently of age.

Clearly any observed association between TL and aPWV in our data relies on the assumption that leukocyte TL reflects the TL of aortic tissue, which may not necessarily be the case. Indeed, a previous study based on post‐mortem tissue (Takubo *et al*. 2002) found no evidence of a cross‐sectional association between age and TL in myocardium or cerebral cortex (probably reflecting the post‐mitotic nature of these tissues), despite significant correlations in other tissues. Two comparatively small studies of aortic tissue biopsies have reported contradictory findings. In 51 individuals aged between 1 month and 80 years, there was a significant age‐associated reduction in aortic TL (Okuda *et al*. 2000). However, in a further study, TL of leukocytes and aortic tissue biopsies were significantly correlated, both in patients undergoing elective abdominal aortic aneurysm repair or controls without aortic disease at the time of cadaveric donation (Wilson *et al*. 2008). However, in this relatively small study population, there was no correlation between either aortic or leukocyte TL and age, although the age‐range of the study population was narrow.

The present study has some limitations. As a result of the cross‐sectional design, it was not possible to examine causal relationships or assess inter‐individual differences in the rate of TL shortening. Moreover, although we consider that our strategy of comparing TL between extremes of aPWV provided a robust approach for evaluating the relationship between cellular and vascular ageing, the use of extreme samples may increase the potential for sampling bias and could overestimate the strength of associations between variables (Salkind, 2010). Furthermore, in adjusting aPWV for differences in blood pressure, we cannot exclude the possibility that the pressure‐dependence of aPWV differs between younger and older individuals. Because of the retrospective nature of the study, it was not possible to measure telomerase activity, which may have been increased to protect the telomeres from oxidative stress, degradation and shortening, as has been suggested in other studies (Brydon *et al*. 2012; Kiecolt‐Glaser *et al*. 2013). Finally, any observed association between TL and aPWV in our data relies upon the assumption that leukocyte TL reflects TL within the aortic wall, which we were unable to confirm in the present study.

In conclusion, we have shown, for the first time, an inverse association between TL and aPWV in younger individuals but a positive association in older individuals. Therefore, our data illustrate that age significantly modifies the relationship between aPWV and TL in healthy individuals. These data suggest that links between cellular and vascular ageing reflect a complex interaction between genetic and environmental factors acting over the whole life‐course and that future studies should take account of this possibility. Future longitudinal studies are therefore required to better understand these factors throughout the life‐course of biological ageing and their implications in understanding CV risk.

## Additional information

### Competing interests

The authors declare that they have no competing interests.

### Author contributions

All haemodynamic data were collected between Cardiff and Cambridge as part of the Anglo‐Cardiff Collaborative Trial. Blood samples were analysed in Addenbrooke's Hospital, University of Cambridge. Telomere samples were analysed in the Biomedical Sciences laboratory, Cardiff School of Health Sciences, Cardiff Metropolitan University. JRC and IBW contributed to the conception or design of the work. BMD, Y, LB, CME, IBW, JE and JRC contributed to the acquisition, analysis or interpretation of data. BMD, Y, LB, CME, IBW, JE and JRC drafted the work or revised it critically for important intellectual content. All authors have approved the final version of the manuscript and agree to be accountable for all aspects of the work in ensuring that questions related to the accuracy or integrity of any part of the work are appropriately investigated and resolved. All persons designated as authors qualify for authorship, and all those who qualify for authorship are listed.

### Funding

Professor Ian B. Wilkinson is a British Heart Foundation Senior Fellow (FS/12/8/29377). Dr Yasmin is supported by the British Heart Foundation (FS/12/8/29377). This work was also supported by the National Institute for Health Research, Cambridge Biomedical Research Centre Award.
